# Colonization and immune modulation properties of *Klebsiella pneumoniae* biofilm-dispersed cells

**DOI:** 10.1038/s41522-019-0098-1

**Published:** 2019-09-24

**Authors:** Cyril Guilhen, Sylvie Miquel, Nicolas Charbonnel, Laura Joseph, Guillaume Carrier, Christiane Forestier, Damien Balestrino

**Affiliations:** 1 Université Clermont Auvergne, CNRS 6023, LMGE, Clermont-Ferrand, France; 2 Université Clermont Auvergne, Inserm U1071, USC-INRA 2018, M2iSH, CRNH Auvergne, Clermont-Ferrand, France; 3 Present Address: Université de Genève, Centre Médical Universitaire, Département de Physiologie Cellulaire et Métabolisme, Genève, Suisse; 4 Present Address: Department of Surgical Oncology, Institut du Cancer de Montpellier, Montpellier, France

**Keywords:** Bacteriology, Biofilms

## Abstract

Biofilm-dispersal is a key determinant for further dissemination of biofilm-embedded bacteria. Recent evidence indicates that biofilm-dispersed bacteria have transcriptional features different from those of both biofilm and planktonic bacteria. In this study, the in vitro and in vivo phenotypic properties of *Klebsiella pneumoniae* cells spontaneously dispersed from biofilm were compared with those of planktonic and sessile cells. Biofilm-dispersed cells, whose growth rate was the same as that of exponential planktonic bacteria but significantly higher than those of sessile and stationary planktonic forms, colonized both abiotic and biotic surfaces more efficiently than their planktonic counterparts regardless of their initial adhesion capabilities. Microscopy studies suggested that dispersed bacteria initiate formation of microcolonies more rapidly than planktonic bacteria. In addition, dispersed cells have both a higher engulfment rate and better survival/multiplication inside macrophages than planktonic cells and sessile cells. In an in vivo murine pneumonia model, the bacterial load in mice lungs infected with biofilm-dispersed bacteria was similar at 6, 24 and 48 h after infection to that of mice lungs infected with planktonic or sessile bacteria. However, biofilm-dispersed and sessile bacteria trend to elicit innate immune response in lungs to a lesser extent than planktonic bacteria. Collectively, the findings from this study suggest that the greater ability of *K. pneumoniae* biofilm-dispersed cells to efficiently achieve surface colonization and to subvert the host immune response confers them substantial advantages in the first steps of the infection process over planktonic bacteria.

## Introduction

*Klebsiella pneumoniae* is an opportunist pathogen ubiquitous in nature and found asymptomatically in more than 40% of the population.^1^ It has been identified as a direct menace for human health owing to its capacity to resist many antibiotics,^2–4^ and its pivotal role in the initial acquisition and spreading of antibiotic-resistant genes.^5^ The high ability of *K. pneumoniae* to form biofilm and thus to colonize tissues and medical devices is also a main factor contributing to the development of healthcare-associated infections.^6,7^ The formation of biofilms on respiratory devices can lead to pneumonia via bacterial dissemination in the lower respiratory tract,^8,9^ with *K. pneumoniae* being responsible for 9.8% of ventilator-associated pneumonia (VAP) cases in intensive care units.^10^

The development of biofilms, i.e., surface-attached bacteria encased in a self-generated matrix,^11^ is classically described as a sequential process involving adhesion of bacteria to the surface, formation of microcolonies, maturation by synthesis of an exopolymeric matrix, and finally dispersal.^12^ Dispersal involves the sensing of signals and their transduction through complex regulatory pathways. It is the necessary step that allows bacteria to leave the biofilm macrostructure as free-living cells able to colonize new locations, hence its prominent role in biofilm-related infections.^13–15^ For instance, *Staphyloccocus epidermidis* mutants deficient in dispersal effectors are less virulent because unable to promote their systemic dissemination from the indwelling device in a mouse model.^16^ Likewise, in vivo dispersal of *P. aeruginosa* biofilm induced by enzymatic agents causes lethal septicemia in a mouse wound model.^17^

Biofilm-dispersed bacteria have long been described as planktonic cells, but a few studies have shown that they possess specific properties. Several recent transcriptional analyses showed that biofilm-dispersed bacteria exhibit specific patterns, distinct from planktonic and sessile lifestyles.^18–20^ A few phenotypic analyses have also shown that they have specific characteristics different from those of planktonic bacteria, such as the overexpression of virulence factors.^18,19,21–23^ For example, *P. aeruginosa* and *S. pneumoniae* biofilm-dispersed bacteria kill epithelial and macrophage cells lines more effectively and have greater virulence in in vivo models than their planktonic counterparts.^18,19,21^ However, apart from these few cases, little is known about the properties of biofilm-dispersed cells. This is partly due to the technical difficulties encountered in harvesting biofilm-dispersed bacteria. For this reason, most studies used signals as inducers of biofilm dispersal.^24,25^ For instance, an increase in temperature causes dispersal of *Staphylococcus aureus* biofilm,^26,27^ and a decrease in cellular c-di-GMP concentration obtained by a chemical or enzymatic approach has been used to disperse *P. aeruginosa* biofilm.^24,25^

In this work, we assessed the intrinsic characteristics of *K. pneumoniae* cells spontaneously dispersed from mature biofilm, concentrating on their metabolic activity and colonization capabilities, and the host-induced immune responses compared to those of their sessile and planktonic counterparts. We were able to show that biofilm-dispersed bacteria are predisposed to efficiently colonize new surfaces and to potentially impair host response.

## Results

### *K. pneumoniae* biofilm-dispersed bacteria have specific physiology

**Fig. 1:**
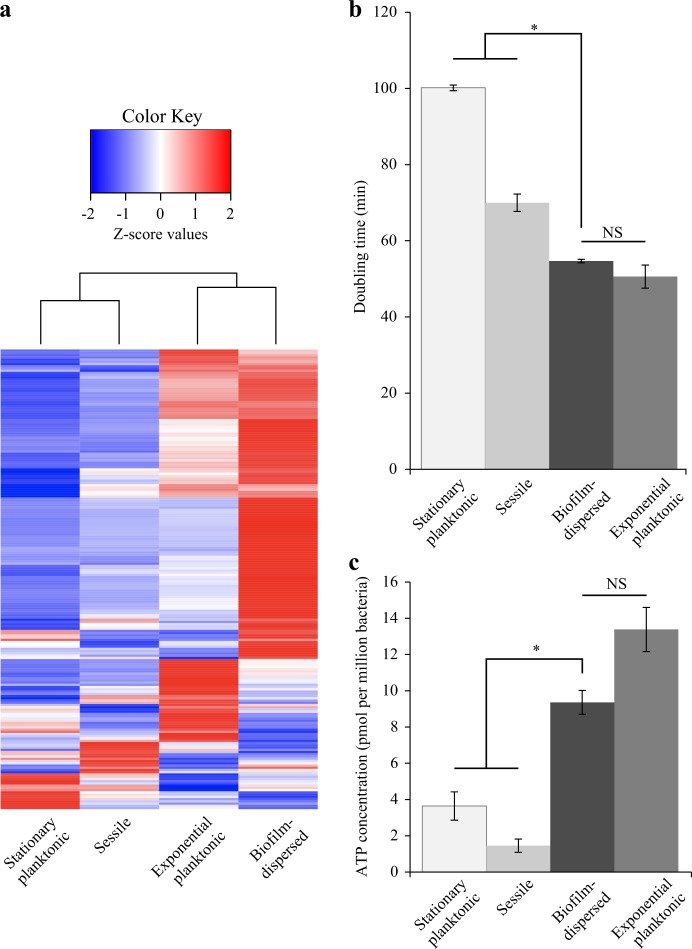
*K. pneumoniae* biofilm-dispersed bacteria have elevated metabolic activity. **a** Heatmap depicting the expression values of the 205 CDS annotated in the COG “Translation, ribosomal structure and biogenesis”. Z-score values, obtained from a published transcriptomic study,^20^ were clustered as columns with hierarchical clustering. **b** Bacteria from the different bacterial states were cultured in fresh medium and the doubling time was obtained on the basis of the slope calculated in accordance with the growth curve of the first hour of culture. Values represent mean ± s.e.m (*n* = 3). **c** Intracellular ATP concentration was monitored in the different bacterial states and normalized by determination of the CFU number. Values represent mean ± s.e.m. (*n* = 3)

**Fig. 2:**
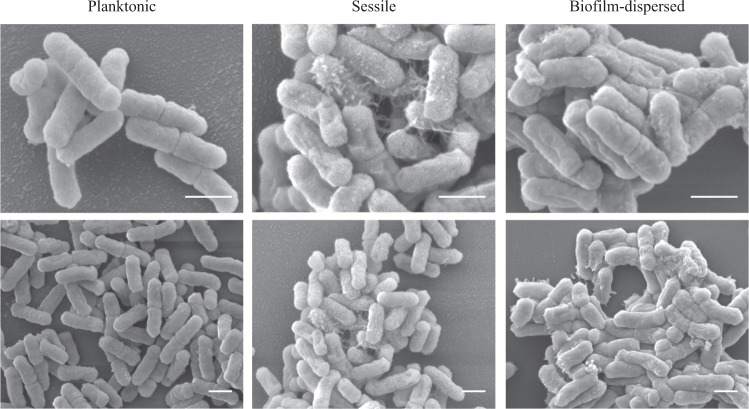
Scanning electron microscopy (SEM) observations of *K. pneumoniae* planktonic and biofilm-dispersed bacteria. Magnifications: ×200,000 upper pictures, ×10,000 lower pictures. Scale bar: 1 µm. The images are representative of the general morphology observed in each case

To assess the metabolic state of biofilm-dispersed cells, we looked at the expression level of the 205 CDS previously annotated in the Clusters of Orthologous Groups (COGs) “Translation, ribosomal structure and biogenesis”.^20^ A large number of them [156] were overexpressed (Z-score > 0) in biofilm-dispersed bacteria compared to planktonic and sessile growth conditions (Fig. 1a). In addition, dispersed bacteria had a growth rate and intracellular pool of ATP similar to those of exponential planktonic cells but significantly higher than those of sessile and stationary planktonic forms, supporting the idea that dispersed cells are particularly metabolically active. Sessile bacteria had the lowest ATP content of all the conditions tested (Fig. 1b, c). Although biofilm-dispersed bacteria had a metabolic activity different from that of sessile bacteria, scanning electron microscopy (SEM) observations showed that biofilm-dispersed and sessile bacteria had an altered morphology and, unlike planktonic cells, were covered by extracellular material (Fig. 2).

### *K. pneumoniae* biofilm-dispersed bacteria are highly efficient in colonizing abiotic and biotic surfaces

**Fig. 3:**
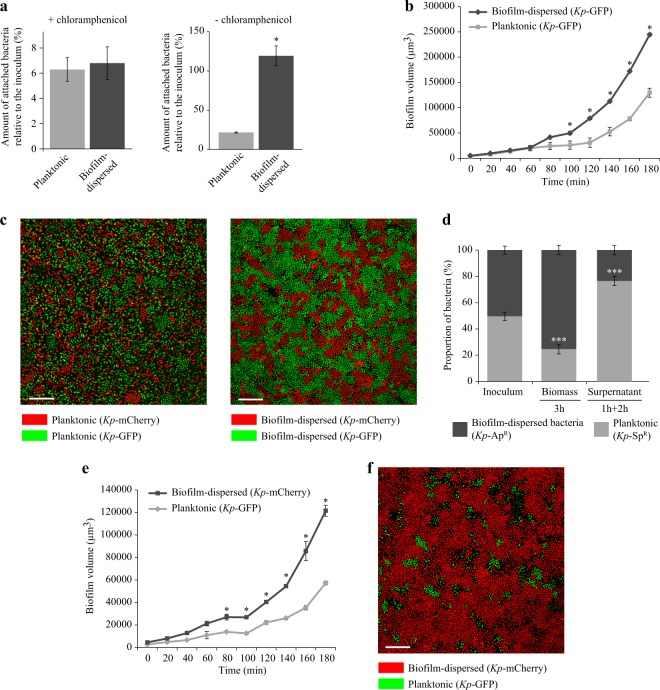
*K. pneumoniae* biofilm-dispersed bacteria efficiently colonize abiotic surfaces and outcompete the planktonic bacteria during surface colonization. **a** The initial adhesion to glass was assessed in the presence of chloramphenicol, which inhibits de novo protein synthesis; left panel. The glass surface colonization was assessed in the absence of chloramphenicol; right panel. Results are presented as the percentages of adherent bacteria after 3 h of incubation compared to the CFU in the inoculum. **b** The kinetics of colonization of the glass surface by *Kp*-GFP was monitored by confocal microscopy. Biofilm volumes were calculated with IMARIS software and are represented in µm^3^. **c** Surface colonization by clonal division of the bacteria was visualized by confocal microscopy after 3 h of inoculation. The surface was seeded with two clones of a given lifestyle, i.e., planktonic or biofilm-dispersed, tagged with different fluorochromes (GFP or mCherry). Images represent the first optical section of the z-stack from the surface after 3 h of incubation. **d** The ability of bacteria to colonize the glass substrate was assessed in a context of competition (biofilm-dispersed bacteria versus planktonic bacteria) and determined by CFU counting of the biomass adhering to the surface after 3 h of incubation. The numbers of free-living bacteria were determined by CFU counting in the supernatant of a 1h-old biofilm, formed by inoculation of equal numbers of biofilm-dispersed (ampicillin resistant) and planktonic bacteria (spectinomycin resistant), further incubated for 2 h. **e** The kinetics of colonization of the glass surface in a context of competition (biofilm-dispersed bacteria versus planktonic bacteria) was monitored by confocal microscopy. Cells derived from biofilm-dispersed and planktonic bacteria were distinguished by their specific fluorescent color. Biofilm volumes were calculated with IMARIS software and are represented in µm^3^. **f** Confocal microscopy observations of the surface coverage by bacteria in a context of competition after 3 h of incubation. The surface was seeded with planktonic and biofilm-dispersed bacteria tagged with GFP and mCherry respectively. Images represent the first optical section of the z-stack from the surface. Scale bar: 20 µm. Values represent mean ± s.e.m. (*n* = 3). Statistics: non-parametric Mann–Whitney test: *p < 0.05; **p < 0.01

To assess whether *K. pneumoniae* biofilm-dispersed bacteria have different adhesion and colonization abilities from those of planktonic bacteria despite their similarities in terms of metabolic activity, we measured the biomass present on a glass surface after 3 h of incubation. Biofilm-dispersed bacteria generated more biomass than planktonic cells (Fig. 3a, right panel). When the assay was performed in the presence of chloramphenicol, a bacteriostatic antibiotic inhibiting de novo protein synthesis, the bacteria had similar adhesion levels irrespective of their initial lifestyle (Fig. 3a, left panel). Analysis of the kinetics of surface colonization by real-time confocal imaging showed that biofilm-dispersed cells generated significantly higher biomass than planktonic cells as early as after 100 min of incubation (Fig. 3b).

To follow the clonal expansion of bacteria during the 3 h initial step of surface coverage, the surface was seeded with two clones of a given lifestyle, planktonic or biofilm-dispersed, tagged with different fluorochromes, either GFP or mCherry. Biofilm-dispersed bacteria formed large microcolonies after 3 h of incubation, whereas planktonic cells formed small microcolonies with lower density surface coverage (Fig. 3c). The nature of the fluorescent protein expressed had no impact on the bacterial kinetics of colonization (Supplementary Fig. 1).

Colonization experiments were also conducted with mixed populations formed with similar inocula of differentially-tagged biofilm-dispersed and planktonic bacteria. CFU quantification of the biomass formed on the glass surface after 3 h of incubation showed that biofilm-dispersed bacteria generated a greater biomass than their planktonic counterparts (Fig. 3d). This difference could not be attributed to autoaggregation properties of the bacteria since both biofilm-dispersed and planktonic bacteria had the same low kinetics of sedimentation in static tube assay (Supplementary Fig. 2). Moreover, flow cytometry analysis of sonicated biofilm-dispersed bacteria showed no differences in size and structure from those of the non-sonicated samples, suggesting that biofilm-dispersed bacteria did not comprise aggregates (Supplementary Fig. 3).

In mixed biofilm, the analysis of the kinetics of surface colonization showed that the biomass from the biofilm-dispersed bacteria outcompeted rapidly that from their planktonic counterparts due to rapid formation of microcolonies (Fig. 3e, f and Supplementary Movie 1). In addition, the number of free-living bacteria in the supernatant of a biofilm derived from biofilm-dispersed cells was lower than that of biofilm derived from planktonic bacteria incubated under the same conditions (Fig. 3d). This result suggests that biofilm-dispersed bacteria stick together and onto the substratum while they multiply whereas planktonic bacteria are released in the supernatant and do not colonize the surface as efficiently.

**Fig. 4:**
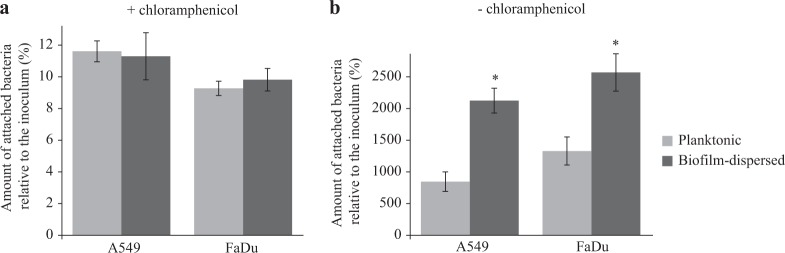
*K. pneumoniae* biofilm-dispersed bacteria efficiently colonize biotic surfaces. **a** The initial adhesion to A549 lung and to FaDu pharyngeal epithelial cells was assessed in the presence of chloramphenicol by CFU determination of adherent bacteria to the monolayers after 3 h of incubation. Results are presented as the percentages of cell-associated bacteria compared to the inoculum. **b** The bacterial colonization of cell monolayers was assessed after 3 h of incubation in the absence of chloramphenicol by CFU counting. Results are presented as the percentages of cell-associated bacteria compared to the inoculum. Each value is the mean of three independent experiments. Values represent mean ± s.e.m. (*n* = 3). Statistics: non-parametric Mann–Whitney test: *p < 0.05; ***p < 0.001

In line with the previous observations on an abiotic surface, bacterial adhesion to pharyngeal (FaDu) and lung (A549) epithelial cells measured in the presence of chloramphenicol was similar for biofilm-dispersed and planktonic bacteria. However, bacteria derived from biofilm-dispersed cells had significantly higher colonization capacities of the cell lawn than their planktonic counterparts (Fig. 4). Altogether, these results suggest that biofilm-dispersed bacteria have intrinsic predispositions to colonize biotic and abiotic surfaces.

### Sessile and biofilm-dispersed *K. pneumoniae* cells induce an attenuated host response compared to planktonic cells

**Fig. 5:**
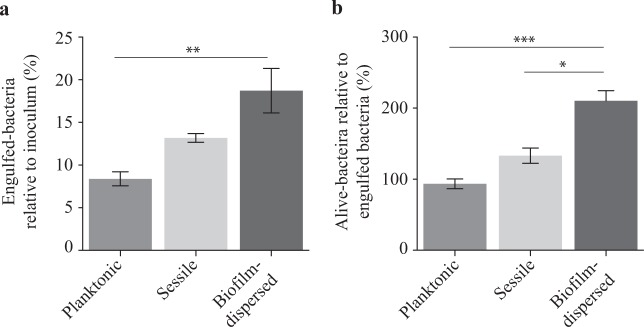
*K. pneumoniae* biofilm-dispersed bacteria display higher engulfment rates by J774A.1 murine macrophages than that of planktonic cells, and a higher survival/multiplication rate than both planktonic and sessile bacteria. **a** Bacterial engulfment by macrophages is expressed as the number of intracellular bacteria at 1 h post-infection relative to the bacterial number in the inoculum. **b** Bacterial survival/multiplication is expressed as the number of intracellular bacteria at 24 h post-infection relative to that obtained after 1 h post-infection. Values represent mean ± s.e.m. (*n* = 3). Statistics: non-parametric Mann–Whitney test: *p < 0.05; ***p < 0.001

The assessment of engulfment within macrophages showed that the internalization of biofilm-dispersed cells by J774A.1 murine macrophages was higher than that of planktonic and sessile bacteria (Fig. 5a). The percentage of alive-bacteria relative to engulfed bacteria after 24 h of incubation, which is the result of both bacterial death and bacterial multiplication, was significantly higher for biofilm-dispersed cells than for planktonic and sessile bacteria (Fig. 5b).

**Fig. 6:**
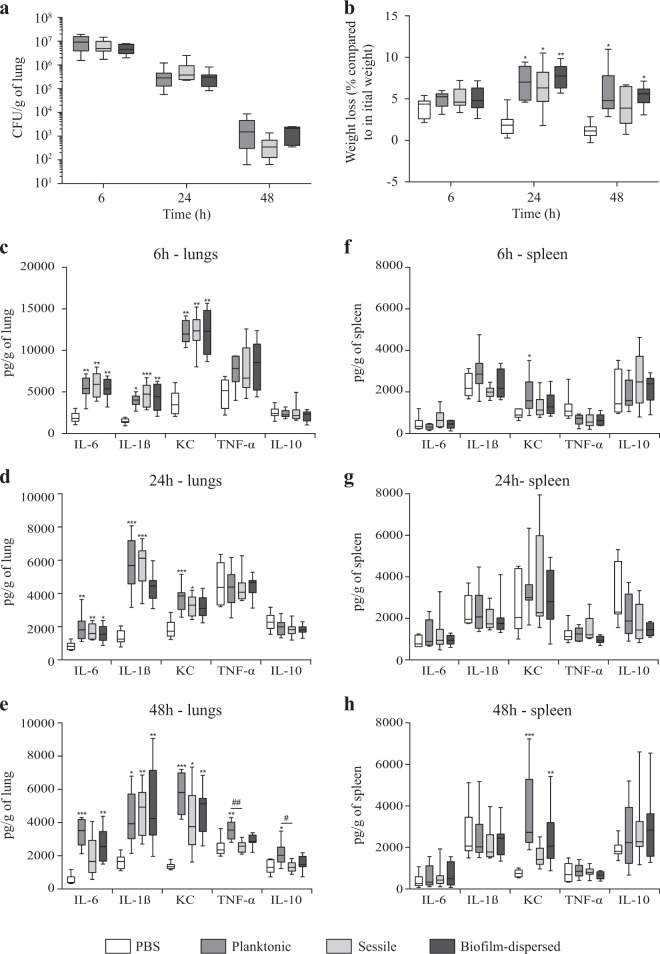
*K. pneumoniae* sessile and biofilm-dispersed bacteria induce an attenuated inflammatory response in a murine model after intra-nasal instillation. **a** Boxplots reflect numbers of *K. pneumoniae* cells recovered in the lungs of animals (*n* = 8) at time points of 6, 24 and 48 h after inoculation of either planktonic, sessile or biofilm-dispersed bacteria. **b** Boxplots reflect weight loss (*n* = 8) 6, 24 and 48 h after intra-nasal instillation of PBS or bacteria. **c**–**e** Lung and **f**–**h** spleen cytokine levels (IL-6, IL-1β, KC, TNF-α, and IL-10) from PBS-treated (white bars), planktonic (medium gray bars), sessile (light gray bars) and biofilm-dispersed (dark gray bars) bacteria infected mice at time points of 6 h (**c** and **f**), 24 h (**d** and **g**) and 48 h (**e** and **h**) after inoculation. Boxplots reflect cytokine levels (*n* = 8). The values are displayed as box-and-whiskers plots with interquartile range, with the top portion of the box representing the 75th percentile, and the bottom portion representing the 25th percentile. The horizontal bar within the box represents the median. Statistics: non-parametric Kruskal–Wallis with Dunn’s multiple comparison test or Tukey’s multiple comparison test for ELISA experiments: *p < 0.05; **p < 0.01; ***p < 0.001 in comparisons performed to the non-infected control; ^#^*p* < 0.05; ^##^*p* < 0.01

In a murine model of lung colonization, similar numbers of viable *K. pneumoniae* were detected in the lungs of mice 6, 24 and 48 h after intra-nasal instillation of either planktonic, sessile or biofilm-dispersed cells (Fig. 6a). No bacteria were detected in spleen for any group of animals whatever the time point of infection. After weighing of the animals, it was observed that all groups of mice receiving bacteria had a significant loss of weight at 24 h time point after inoculation compared to the control group treated with PBS (Fig. 6b). However, 48 h after inoculation, the difference was not statistically significant in the group of animals inoculated with sessile bacteria (Fig. 6b). To assess the innate host inflammatory response to infection, IL-6, IL-1β, KC, TNF-α, and IL-10 levels were measured in lung and spleen homogenates, and in the blood. No detectable level of these cytokines was measured in blood samples of the animals. Whatever the bacterial lifestyle, i.e., planktonic, sessile or biofilm-dispersed, *K. pneumoniae* induced production of the pro-inflammatory cytokines IL-6, IL-1β, and KC in the lungs as early as 6 h post-infection. The levels of these cytokines in the lungs at 6 h time point after inoculation were higher than those measured after 24 h and 48 h of infection (Fig. 6c–e). The levels of these pro-inflammatory cytokines in infected animals were always maintained significantly higher than those measured in the non-infected mice during the 48 h period of the assays (Fig. 6c–e), except for the IL-1ß and KC levels in mice infected with biofilm-dispersed bacteria which were not significantly different than those of the non-infected mice at the 24 h time point after infection (Fig. 6b). Moreover, the pro-inflammatory response was less extensive with sessile bacteria than with planktonic bacteria at 48 h post-infection (Fig. 6e). At this time point, all animals infected with sessile *K. pneumoniae* had a significant lower production of TNF-α and IL-10 in the lungs than those infected with planktonic bacteria (Fig. 6e). The biofilm-dispersed bacteria induced an intermediary immune response compared to those induced by planktonic or sessile bacteria, lower than that induced by planktonic bacteria and higher than that induced by sessile bacteria (Fig. 6c–e).

In spleen, no cytokine response was observed, except for the pro-inflammatory marker KC that was significantly increased at the 48 h time point in the groups of mice inoculated with planktonic and biofilm-dispersed bacteria compared to the control group (Fig. 6f–h). Although significant, the increase of KC level in mice infected with biofilm-dispersed bacteria was lower than that of mice infected with planktonic bacteria (Fig. 6h). The TNF-α /IL-10 ratios were not significantly changed regardless of the organ, the bacteria lifestyle and the time point post-inoculation, except in the spleen of mice infected with sessile bacteria where they were significantly lower than in non-infected mice at 24 h time point after infection (Supplementary Fig. 4).

## Discussion

Biofilm dispersal is a critical step in the colonization of new environmental niches. It is particularly problematic in medicine since it is involved in the development of systemic infections by promoting the transition from biofilm colonization to dissemination and invasive disease.^15,16^ Dispersal can be triggered by host signals, as observed in the pathophysiology of secondary pneumonia due to *Streptococcus pneumoniae* and *S. aureus*, or can be considered as the normal physiology of biofilm when intrinsically induced.^15,21,26^ Disseminated bacteria encounter various potentially stressful environments such as the host immune system and antimicrobial agents and have probably developed strategies to escape from or fight against these threats.^15^ Several studies, especially transcriptomic analyses, have suggested that biofilm-dispersed cells have a specific phenotype, different from that of planktonic and sessile bacteria.^18–23^ In this study, we investigated the colonization abilities of *K. pneumoniae* biofilm-dispersed cells in different models and their interactions with the host immune system.

Data from our study showed that *K. pneumoniae* biofilm-dispersed bacteria had enhanced colonization capabilities, i.e., adhesion coupled with bacterial multiplication on a substratum, on both biotic and abiotic surfaces. These properties could be due to high metabolic activity but *K. pneumoniae* dispersed bacteria had growth and ATP content similar to those of planktonic bacteria, as previously observed with *S. pneumoniae*.^18^ A second hypothesis would be that *K. pneumoniae* biofilm-dispersed cells possess specific physico-chemical surface properties that enable the cells to rapidly adhere to the substrates. Berlanga et al.^28^ recently showed that the surface of *Klebsiella oxytoca* biofilm-dispersed cells was more hydrophobic than that of their planktonic counterparts, thereby facilitating adhesion to polystyrene and the biofilm formation process on similar surfaces. In our study, the initial adhesion capabilities of the *K. pneumoniae* biofilm-dispersed cells were similar to those of their planktonic counterparts, suggesting that no adhesin was specifically exposed at the surface of biofilm-dispersed bacteria, and there was no difference in their surface physicochemical properties as assessed by microbial affinity to solvents (MATS) test (Supplementary Table 1). A third hypothesis is that dispersed cells consist of small aggregates, which would thus accelerate the process of colonization, as described with *P. aeruginosa*.^29,30^ However, in our study the *K. pneumoniae* dispersal population had no multicellular aggregates, the autoaggregation phenotype was similar in biofilm-dispersed and planktonic lifestyles (Supplementary Fig. 2) and disruption of the potential aggregates by sonication had no impact on the colonization capabilities of biofilm-dispersed bacteria (Supplementary Fig. 5). The efficient ability of *K. pneumoniae* dispersed cells to colonize new surfaces would therefore be due to a specific capacity of such cells to rapidly form microcolonies, as observed by microscopic examination. The presence of potential residues at the surface of dispersed bacteria (Fig. 2) may enhance the formation of microcolonies thereby promoting biofilm formation and giving them a selective advantage over planktonic cells (Fig. 3e, f). Since efficient surface colonization by biofilm-dispersed bacteria requires protein de novo biosynthesis, we can assume that dispersed bacteria do not have specific adhesins overexpressed on the bacterial surface when they leave the biofilm biomass. The initial colonization step would be ensured by rapidly synthetized components, such as matrix components or adhesins. Accordingly, previous transcriptional analyses showed that several operons involved in colonization and biofilm formation, such as the *kpg* and *kpj* operons, are overexpressed in biofilm-dispersed bacteria compared to planktonic bacteria.^20,31^

*K. pneumoniae* biofilm-dispersed cells could also possess specific properties that enable them to escape the immune system during interactions with the host defenses. However, *K. pneumoniae* biofilm-dispersed bacteria were phagocytosed at a higher level than planktonic bacteria, unlike *P. aeruginosa* biofilm-dispersed bacteria, which have a greater ability to escape from engulfment by macrophages than their planktonic counterparts.^19^ *K. pneumoniae* expresses capsule on its surface, which impairs phagocytosis.^32^ However, RNA sequencing data showed that the expression of the capsule-encoding genes is similar in *K. pneumoniae* planktonic and biofilm-dispersed bacteria^20^ and therefore cannot account for the modulation of engulfment of biofilm-dispersed cells by macrophages. It cannot be excluded however that capsule shedding is different between the populations and may explain the differences in uptake. Moreover, the enhanced engulfment of biofilm-dispersed bacteria could be due to other mechanisms, such as the interaction of bacterial moieties specifically expressed by biofilm-dispersed bacteria with specific macrophage receptors. In contrast, the *K. pneumoniae* sessile population, consisting of individualized bacteria, showed a level of phagocytosis not significantly different from that of planktonic bacteria. Likewise, *S. epidermidis* singularized sessile bacteria were taken up by J774A.1 macrophages as efficiently as the free-living form, whereas the biofilm organization impaired phagocytosis.^33^

In our study, biofilm-dispersed bacteria were recovered at a higher level than planktonic and sessile bacteria after 24 h of incubation in macrophages owing to a higher resistance to bactericidal activity after uptake and/or a higher multiplication rate inside phagocytes. Cano et al.^34^ reported that *K. pneumoniae* is able to interfere with phagosome maturation by avoiding fusion with lysosomes. The pathogen-associated molecular patterns (PAMPs) of microbes engulfed within macrophages can influence, positively or negatively, the kinetics of phagosome maturation (for review see ref. ^35^). The presence of specific residues at the surface of *K. pneumoniae* biofilm-dispersed bacteria could impair phagosome maturation to a greater extent than with planktonic cells. Nor can we exclude the possibility that *K. pneumoniae* biofilm-dispersed bacteria are able to counteract intracellular intoxication by phagosome products. The high copper content of macrophage phagolysosomes plays an important part in toxicity against several pathogens (for review see ref. ^36^) and some of them are equipped with a battery of copper detoxification defenses. Interestingly, in *K. pneumoniae* biofilm-dispersed bacteria, the two *cusABFC* operons related to copper efflux are overexpressed (in one of them more than 100-fold) compared to their levels of expression in planktonic bacteria.^20^ Future studies are warranted to assess the underlying mechanisms that allow *K. pneumoniae* dispersed bacteria to efficiently survive in macrophages.

Several studies have also reported the pathogenicity of biofilm-dispersed bacteria in animal models. For instance, intraperitoneal inoculation of biofilm-dispersed pneumococcal cells in a murine septicemia model showed that they were more virulent than their planktonic and sessile counterparts.^18^ To better characterize the behavior in vivo of *K. pneumoniae* spontaneously biofilm-dispersed bacteria, we assessed their virulence in an integrative model of murine pneumonia by measuring the lung colonization and the cytokine response in the lungs and spleen. No significant difference was observed in the number of bacteria recovered at the different time-points post-infection in the animals’ lung tissues inoculated with either sessile, biofilm-dispersed bacteria or their planktonic counterparts. Surprisingly, no bacteria were recovered in the spleen, whatever the times point of infection, contrary to previous studies showing bacterial dissemination from lungs.^37,38^ The levels of the pro-inflammatory cytokines IL-1β, KC, and IL-6 detected in the lungs of infected animals were higher than in the non-infected animals. At the 48 h time point, an increased production of the pro-inflammatory TNF-α was observed only in animals infected by planktonic bacteria, suggesting a lower elicitation of the innate immune system by sessile and biofilm-dispersed bacteria. This finding is of particularly interest since biofilm-dispersed bacteria are those probably able to migrate during *K. pneumoniae* pulmonary infection. The lower elicitation of the immune system by sessile and biofilm-dispersed bacteria is however unlikely to impact the bacterial burden in lungs, at least in our model as demonstrated by measurement of the bacterial loads.

Although no study has previously analyzed the host response to biofilm-dispersed bacteria, the sessile phenotype has already been shown to accentuate the anti-inflammatory response induced by some bacterial species, especially *Lactobacillus*.^39^ However, the immune response triggered by biofilms is complex since biofilms can both suppress and overstimulate the immune system, depending on the immune status of the host or the bacterial species composition of the biofilm.^40^

Although *Klebsiella* can counteract the activation of inflammatory responses by attenuating pro-inflammatory Il-1β-induced IL-8 expression,^41,42^ it usually induces the production of pro-inflammatory cytokines in such murine lung infection models.^37,41^ Siderophore production has recently been described as a major trigger of inflammation and bacterial dissemination during lung infection.^37^ Interestingly, the expression of all siderophores encoding genes, including those encoding yersiniabactin, enterobactin, salmochelin and aerobactin, are greatly diminished (up to more than 1000-fold for the yersiniabactin *ybtPQXS* genes encoding the transport system) in *K. pneumoniae* biofilm-dispersed and sessile cells compared to planktonic cells.^20^ This suggests that biofilm-dispersed and sessile bacteria down-regulate siderophore expression to hide from the immune system and avoid a strong proinflammatory response.

Several studies have shown that biofilm-dispersed bacteria had greater pathogenicity than their planktonic counterparts.^15^ Our results are in agreement with the notion that *K. pneumoniae* biofilm-dispersed bacteria, like sessile bacteria, trend to elicit a lower inflammation than planktonic bacteria, an essential property for pathogen survival during infection. In addition, biofilm-dispersed bacteria have metabolic activity as high as that of planktonic free-living bacteria, but a greater ability to colonize surfaces. Our results are consistent with the notion that *K. pneumoniae* biofilm-dispersed bacteria could have a potential advantage in the initial step of the infection process. Hence, therapies aiming to disrupt biofilms by promoting their dispersal warrant careful consideration. A thorough understanding of the physiology of biofilm-dispersed bacteria is essential to develop new strategies combining the use of a dispersal signal and antibacterial agents that target potential weaknesses of biofilm-dispersed bacteria.

## Methods

### Bacterial strains, plasmids, and culture conditions

The bacterial strains and plasmids used in this study are given in Table 1. *K. pneumoniae* strains were grown in 0.4% glucose M63B1 minimal medium (M63B1) at 37 °C and stored in Lysogeny Broth (LB) broth containing 15% glycerol at −80 °C. Planktonic bacteria were cultured under aerobic conditions and harvested at OD_620_ = 0.8 (exponential phase of growth) or after overnight growth (stationary phase of growth). Sessile and biofilm-dispersed bacteria were harvested from a flow-cell device as described in detail in.^20^ Briefly, a glass cover slip ensuring a surface for biofilm development was glued with silicon glue (3M, Saint Paul, Minnesota, USA) on a flow-cell with one chamber (dimension: 54 mm × 19 mm × 6 mm; 6156 mm^3^). Before experiments, the system was sterilized by pumping 10% (wt/vol) hypochlorite sodium for 1 h and then ethanol 100% (vol/vol) for 15 min. Thereafter, the system was rinsed with a continuous flow of M63B1 medium for 2 h at 37 °C. The inoculum, composed of 10^8^ CFU from an overnight culture of *K. pneumoniae*, was injected with a syringe into the flow-cell. After 1 h of incubation at 37 °C without flow to allow bacterial adhesion, M63B1 medium was pumped at a constant rate of 0.9 mL/min through the device. Sessile and biofilm-dispersed bacteria were recovered after 16 h of culture in the flow-cell or its effluent, respectively. The sessile bacteria were prepared by disrupting the biofilm by vortex; no cell clumps were detected by optical microscopy. All samples were kept on ice until use.

**Table 1:** Strains and plasmids used in this study

Strain or plasmid	Description	Antibiotic resistances	Collection number	Source and/or reference
Strains				
* K. pneumoniae*				
LM21-GFP-Sp^R^	*K. pneumoniae* LM21 strain harboring the *gfpmut3* and *aadA7* genes	Sp	CH404	^46^
* Kp*-Ap^R^	*K. pneumoniae* clinicate isolate	Ap	CH1157	This study
* Kp*-Sp^R^	*Kp*-Ap^R^ Δ*shv::aadA7*	Sp	CH1476	This study
* Kp*-GFP	mini-Tn7T-Km-*GFPmut3* inserted into *att*Tn7 sites of *Kp*-Sp^R^	Sp, Km	CH1477	This study
* Kp*-mCherry	mini-Tn7T-Km-*cCherry* inserted into *att*Tn7 sites of *Kp*-Sp^R^	Sp, Km	CH1478	This study
* E. coli* K-12				
MFD λpir	MG1655 RP4-2-Tc::[∆Mu1::*aac*(3)*IV-∆aphA-nic35*-Mu2::*zeo*]∆*dapA*::(*erm-pir*) ∆*recA*	Ap, Zeo, Erm		^47^
Plasmids				
pTNS3	Helper plasmid, providing the Tn7 transposition function. R6K *ori*, *oriT*	Ap		^48^
pUC18R6KT-mini-Tn7T-Km	pUC18-based delivery plasmid for mini-Tn7-Km. R6K *ori*, *oriT*	Ap, Km		^48^
pUC18R6KT-mini-Tn7T-Km-*gfp*	pUC18-based delivery plasmid for mini-Tn7-Km-*gfp* harboring GFP encoding gene. R6K *ori*, *oriT*	Ap, Km		This study
pUC18R6KT-mini- Tn7T-Km-*mcherry*	pUC18-based delivery plasmid for mini-Tn7-Km-*mcherry* harboring mCherry encoding gene. R6K *ori*, *oriT*	Ap, Km		This study

*Ap* ampicilin, *Sp* spectinomycin, *Km* kanamycin, *Zeo* Zeocine, *Erm* erythromycin

### Construction of *K. pneumoniae*-tagged strains

Strain *Kp*-Sp^R^ was constructed by replacing the *shv-1* β-lactamase-encoding gene (chromosomal ampicillin resistance gene) by *aadA7* (spectinomycin resistance) in strain *Kp*-Ap^R^.^43,44^ The strategy was to replace the chromosomal sequence with a PCR fragment containing the spectinomycin resistance *aadA7* gene amplified from *K. pneumoniae* LM21-GFP-Sp^R^ chromosomal DNA. *Kp*-Sp^R^ was then tagged by insertion of GFP- or mCherry-encoding genes on the chromosome using a mobilizable mini-Tn7 base vector.^45^ Briefly, the *gfpmut3* gene amplified from *K. pneumoniae* LM21-GFP-Sp^R^ genomic DNA, and the codon optimized *mcherry* gene synthetically synthetized (GeneCust, Luxembourg) were cloned under the control of the constitutive λ*p*_R_ promoter in the plasmid pUC18R6KT-mini-Tn7T-Km,^45^ generating pUC18R6KT-mini-Tn7T-Km-*gfp* and pUC18R6KT-mini-Tn7T-Km-*mcherry* plasmids, respectively. Mini-Tn7 delivery was accomplished by three parental matings involving *E. coli* MFD*pir*^46^ harboring the respective mini-Tn7 delivery plasmid, the *K. pneumoniae* recipient strain, and *E. coli* MFD*pir*/pTNS3 encoding the *tnsABCD* genes necessary for the transposition of mini-Tn7 at the *att*Tn7 insertion site.^47^

### Cell lines and culture conditions

The cell lines used in this study were maintained in an atmosphere containing 5% CO_2_ at 37 °C in the culture medium recommended by ATCC. Human epithelial lung A549 cells (ATCC, CCL-185) and murine macrophages J774A.1 (ATCC, TIB-67) were cultured in DMEM high glucose medium (Dominique Dutscher, Brumath, France) supplemented with 10% of heat-inactivated fetal bovine serum (Dominique Dutscher). Human pharyngeal epithelial FaDu cells (ATCC, HTB-43) were cultured in MEM medium (Dominique Dutscher) supplemented with 10% of heat-inactivated fetal bovine serum.

### Quantification of intracellular ATP by enzymatic assay

Intracellular ATP concentrations were determined with planktonic, sessile and biofilm-dispersed *Kp*-Sp^R^ using the ATP determination kit (Invitrogen, Carlsbad, California, USA) according to the manufacturer’s instructions. Briefly, bacteria were washed in PBS and approximatly 5.10^7^ colony-forming units (CFU) were lysed by sonication (10 min – cycles of 30 s ON and 30 s OFF) (Bioruptor® – Diagenode, Liege, Belgium) in the presence of 0.5% Triton X-100 (Euromedex, Souffelweyersheim, France). Cellular extracts were centrifuged at 10,000 × *g* for 1 min, and supernatants were harvested and kept on ice until ATP quantification. In parallel, the initial bacterial load of each sample was quantified by CFU counting to determine the ATP load per bacterial cell.

### Bacterial adhesion and colonization to abiotic surface

For determination of initial bacterial adhesion and bacterial colonization of abiotic surface, round glass coverslips were inoculated in wells of a 24-well plate with 10^7^ CFU of *Kp*-Sp^R^. Initial bacterial adhesion was determined after 3 h of incubation at 37 °C in the presence of chloramphenicol (35 µg/mL), which inhibits de novo protein synthesis. Bacterial colonization was determined in the same manner but in the absence of chloramphenicol. The coverslips were removed, rinsed with PBS, transferred into tubes containing 2 mL of PBS and sonicated for 3 × 5 min. The resulting suspensions were then serially diluted and plated onto LB agar to determine the CFU number. The same procedure was used to evaluate bacterial colonization abilities in a competitive environment, but the inoculum was composed of equal proportions (10^7^ CFU) of biofilm-dispersed *Kp*-Ap^R^ and planktonic *Kp*-Sp^R^ bacteria, resistant to ampicillin and spectinomycin, respectively. To determine the numbers of bacteria released in the supernatant during surface colonization, wells of a 24-well plate containing glass coverslips were inoculated with equal proportions (10^7^ CFU) of biofilm-dispersed *Kp*-Ap^R^ and planktonic *Kp*-Sp^R^ bacteria. After 1 h of incubation at 37 °C, the glass coverslips were rinsed with PBS and placed in new plates containing 1 mL of fresh M63B1 medium and incubated at 37 °C for 2 further hours. The proportion of each bacterium inside the biofilm biomass and in the supernatant was determined by plating onto LB agar containing either ampicillin or spectinomycin.

Bacterial colonization of glass substrates was also investigated by confocal microscopy. A total of 10^7^ CFU were inoculated per chamber on a Lab-Tek^®^ 8 chamber glass slide (Nunc - Thermo Fisher Scientific, Waltham, Massachusetts, USA). In the context of competition, the inoculum was composed of 10^7^ CFU of *Kp*-GFP and *Kp*-mCherry in equal proportions. Biofilm development was monitored at 37 °C for 3 h in real time (one acquisition every 20 min) with a SP5 confocal laser microscope (Leica, Wetzlar, Germany) using ×40 oil objective. GFP was excited by an argon laser (488 nm) and mCherry with the DPSS 561 laser (561 nm). Images represent the first optical section of the z-stack from the surface. Biofilm volumes were calculated with IMARIS software (Bitplane, Belfast, United Kingdom). Each confocal microscopy image is representative of three independent experiments.

### Adhesion to and colonization of epithelial cells

A549 and FaDu epithelial cell monolayers were seeded in 24-well tissue culture plates (Falcon – Corning, New York, USA) at a density of 5 × 10^5^ cells per well on the day before the experiments. Cells were infected with biofilm-dispersed and planktonically growing *Kp*-Sp^R^ such that the multiplicity of infection was 1 bacterium per cell (MOI_1_). To determine bacterial adhesion, chloramphenicol at 35 µg/mL was added to the culture medium to inhibit bacterial growth. After 3 h of incubation, cells were washed two times with PBS, and lysed with 1% Triton X-100 in PBS. Samples were serially diluted, plated onto LB agar plates, and CFUs were determined.

### Analysis of engulfment and degradation by macrophages

For bacterial engulfment and survival/multiplication within macrophages, J774A.1 monolayers were seeded in 24-well tissue culture plates (Falcon – Corning) at a density of 5.10^5^ cells per well on the day before the experiments. Cells were then infected with *Kp*-Sp^R^ resuspended in cell culture medium without antibiotics at a MOI_100_. Infected monolayers were centrifuged at 1000 × *g* for 10 min at 25 °C and then incubated for 20 min at 37 °C. Cells were washed twice with PBS, and fresh cell culture medium containing gentamicin at 50 µg/mL was added for a 30 min (initial engulfment) or 24 h period (survival/multiplication). The numbers of intracellular bacteria were determined by CFU counting after washing cell monolayers twice with PBS and lysing them with 1% Triton X-100 in PBS. Bacterial engulfment was expressed as the mean percentage of bacteria recovered relative to the number of bacteria in the inoculum, defined as 100%. Bacterial survival/multiplication was expressed as the mean percentage of bacteria recovered at 24 h post infection relative to the number of engulfed bacteria, defined as 100%.

### Analysis of the morphology of biofilm-dispersed bacteria

For SEP observations, *Kp*-Sp^R^ bacteria were washed in PHEM rinsing buffer (PIPES: 60 mM; HEPES: 25 mM; EGTA: 10 mM; MgCl_2_: 2 mM) and fixed for 12 h at 4 °C in PHEM buffer, pH 8, which contained 0.05% red ruthenium and 1.6% glutaraldehyde. Samples were then washed 3 × 10 min in PHEM buffer 1X, pH 8. They were post-fixed 1 h with 1% osmium tetroxide in PHEM buffer 1X, pH 8 and washed for 20 min in distilled water. Dehydration with graded ethanol was performed from 25 to 100% (10 min each) and finished in hexamethyldisilazane for 10 min. Bacteria were deposited on Thermanox slides^®^ and dried under a fume hood overnight. The samples were then mounted on stubs with adhesive carbon tabs and sputter-coated with gold-palladium (JFC-1300, JEOL, Japan). Morphology analysis was carried out with a JSM-6060LV (JEOL) scanning electron microscope at 5 kV in high-vacuum mode.

The absence of bacterial aggregates in biofilm-dispersed population was determined by flow cytometry (BD Biosciences, Franklin Lakes, New Jersey, USA). Briefly, *Kp*-Sp^R^ biofilm-dispersed bacteria were centrifuged at 6000 × *g* at 4 °C for 5 min and the pellet was washed once and resuspended in PBS to a final concentration of 10^6^ CFU/mL. Bacteria were labeled with DAPI (0.5 µg/mL), and 50,000 DAPI-positive events were analyzed through the cytometer. Cell size and granularity were assessed by FCS and SSC measurements, respectively.

### Mouse infections and cytokine analysis

Male C57BL/6J wild-type mice aged around 8 weeks were obtained from Charles River Laboratory (SAINT GERMAIN NUELLES, France). Mice strains were bred in a biosafety level 2 animal facility and raised and housed under standard conditions with air filtration. For infection experiments, the mice were housed in cages ventilated under negative pressure with high-efficiency particulate filtered air. Animal protocols were carried out in strict accordance with the recommendations of the Guide for the Care and Use of Laboratory Animals of the University Clermont Auvergne (France) and were approved by the Committee for Research and Ethical Issues of the Department of Auvergne (C2EA-02) following international directive 86/609/CEE (n°CE16-09), and the Ministère de l’Education Nationale, de l’Enseignement Supérieur et de la Recherche (APAFIS#6177-2016072110437265). Mice were anesthetized by a mixture of ketamine and xylazine (1 and 0.2 mg per mouse, respectively) and infected with 1.10^6^ CFU/mouse by intranasal (IN) inoculation. Experiments were done with at least seven animals for each group. Body weight was monitored before IN inoculation and before sacrifice at time points of 6, 24 or 48 h after IN. Mice were sacrificed by cervical dislocation after 6, 24 or 48 h of IN inoculation, and the organs (lungs and spleen) were collected, weighed, and homogenized in sterile PBS. Bacterial load in the organs was determined by plating dilutions of the suspensions on LB agar for CFU quantification. Results are expressed as CFU/g of tissue. For cytokine quantification, tissues homogenates were centrifuged at 13,500 × *g* for 10 min, and levels of IL-6, IL-1β, KC, TNF-α, and IL-10 were assessed in the supernatant with the DuoSet Kit for mice (R&D Systems) according to the manufacturer’s instructions. Results are expressed as pg/g of tissue.

### Statistical analysis

Mann–Whitney and one-way ANOVA (Kruskal–Wallis with Dunn’s Multiple Comparison Test or Tukey’s Multiple Comparison Test) tests were performed with Graph Pad Prism 6 software. A *p*-value less than 0.05 was considered statistically significant. *p < 0.05; **p < 0.01; ***p < 0.001.

### Reporting summary

Further information on research design is available in the Nature Research Reporting Summary linked to this article.

## Supplementary information

Reporting Summary Checklist

Supplementary information.

Supplementary Movie 1.

## Data Availability

All data generated or analyzed during this study are available upon request to the corresponding authors

## Supplementary information

**Supplementary information** accompanies the paper on the *npj Biofilms and Microbiomes* website (10.1038/s41522-019-0098-1).
