# Supplementary material for: Colonization and immune modulation properties of Klebsiella pneumoniae biofilm-dispersed cells
Source: NPJ Biofilms Microbiomes. 2019 Sep 24;5:25. doi: 10.1038/s41522-019-0098-1 (PMC6760147; doi:10.1038/s41522-019-0098-1)
Supplement: Supplementary file 2 — Supplementary information. [file 41522_2019_98_MOESM2_ESM.pdf]

Supplementary information

Colonization and immune modulation properties of *Klebsiella pneumoniae* biofilm-dispersed cells

Authors: Cyril Guilhen,[a*] Sylvie Miquel,[a] Nicolas Charbonnel,[a] Laura Joseph,[a] Guillaume Carrier,[b*] Christiane Forestier,[a] Damien Balestrino[a#]

Affiliations:

[a]Université Clermont Auvergne , CNRS 6023, LMGE, Clermont-Ferrand , France

[b]Université Clermont Auvergne , Inserm U1071, USC-INRA 2018, M2iSH, CRNH Auvergne, Clermont-Ferrand , France

[#]Correspondence to: Damien Balestrino, damien.balestrino@uca.fr

*Present address:

Cyril Guilhen, Université de Genève, Centre Médical Universitaire, Département de Physiologie Cellulaire et Métabolisme, Genève, Suisse

Guillaume Carrier, Institut du Cancer de Montpellier, Montpellier, France

Supplementary Methods

Autoaggregation test. Exponential planktonic and biofilm-dispersed bacteria suspensions were adjusted to OD_{620}= 0.5, and 3-ml portions of each culture were transferred to 5-ml tubes. The tubes were incubated without agitation at room temperature. The OD_{620} of the upper part of the suspension in each standing tube was determined after 4h and 24h of incubation.

MATS assay. The Microbial Adhesion To Solvents (MATS) test was performed to evaluate the Lewis acid-base properties and the hydrophilic/hydrophobic nature of bacterial surfaces. Biofilm-dispersed and exponential planktonic bacteria were washed twice with PBS at pH 7.0, and resuspended in the same solution at a final OD_{620} of 0.5. Each bacterial suspension (1mL) was mixed for 1min at maximum intensity on a vortex-type agitator with 0.2 ml of chloroform, hexadecane, diethyl ether, hexane, ethyl acetate or decane. The mixtures were allowed to stand for 1h to ensure complete separation of the two phases. A 0.8mL sample was then carefully removed from the aqueous phase and its OD_{600} was measured. The microbial affinity for each solvent was calculated using the formula: % affinity for the solvent = ($OD_{(T0)}$- $OD_{(T1h)}$/$OD_{(T0)}$) x 100 where $OD_{(T0)}$ is the optical density of the bacterial suspension before its mixture with the solvent and $OD_{(T1h)}$ the absorbance after mixing and phase separation. Each experiment was performed in triplicate. Three solvent pairs were assayed: (i) chloroform and hexadecane, (ii) diethyl ether and hexane, and (iii) ethyl acetate and decane.

Supplementary Figures

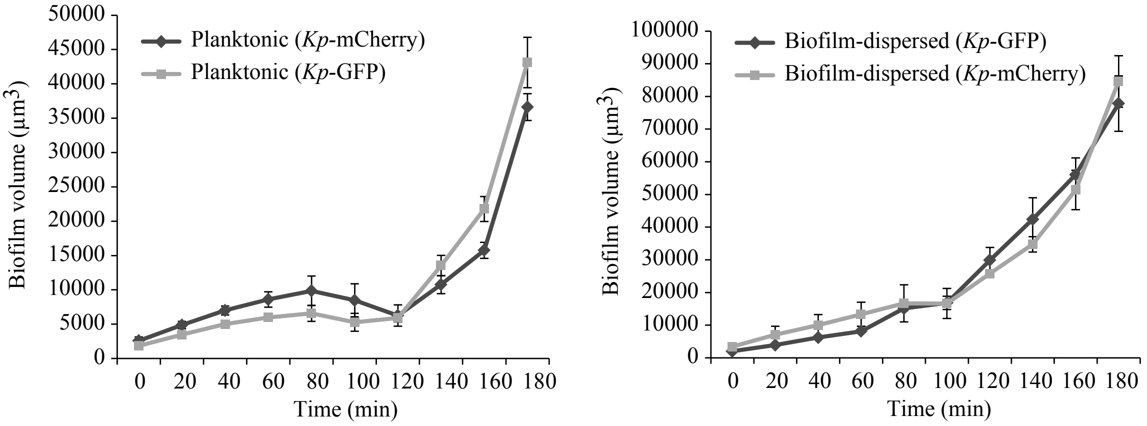

Supplementary Figure 1: The nature of the fluorescent protein had no impact on the bacterial kinetics of colonization. Lab-Tek® glass surface was seeded at T0 with equal numbers of two clones of a given lifestyle, either planktonic or biofilm-dispersed, tagged with different fluorochromes (GFP or mCherry). Their colonization abilities were assessed during 3h of incubation by confocal imaging. Biofilm volumes were calculated with IMARIS software and are represented in μm^3. The different bacterial populations were distinguished by their differential fluorochromes (GFP or mCherry). Values represent mean ± s.e.m ($n = 3$). Statistics: non-parametric Mann-Whitney test: *, $p < 0.05$.

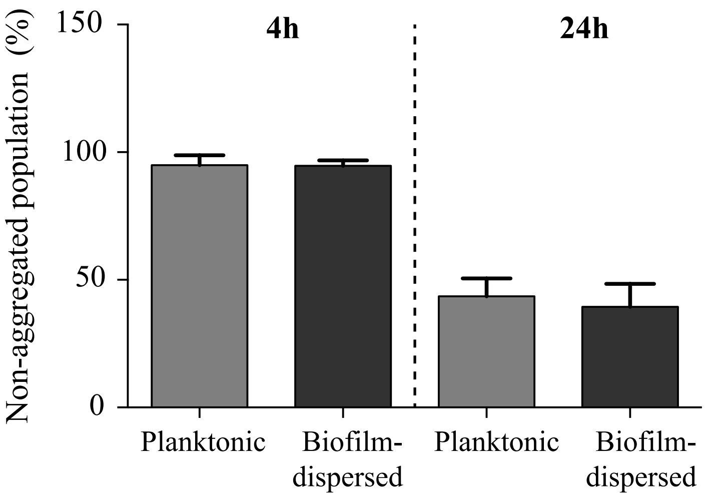

Supplementary Figure 2: Autoaggregation phenotype was similar in biofilm-dispersed and planktonic lifestyles. Autoaggregation assay was performed with exponential planktonic and biofilm-dispersed bacteria. Bacterial suspensions were adjusted to $OD_{620} = 0.5$ in M63B1 in a 3 ml volume and autoaggregation of each suspension over 4h and 24h at room temperature was evaluated by measuring the OD_{620} of the upper part of the culture in each standing tube. Values represent the (OD_{620} at the indicated time point / OD_{620} at T0) x 100. Results are expressed as mean ± s.e.m ($n = 4$). Statistics: non-parametric Mann-Whitney test.

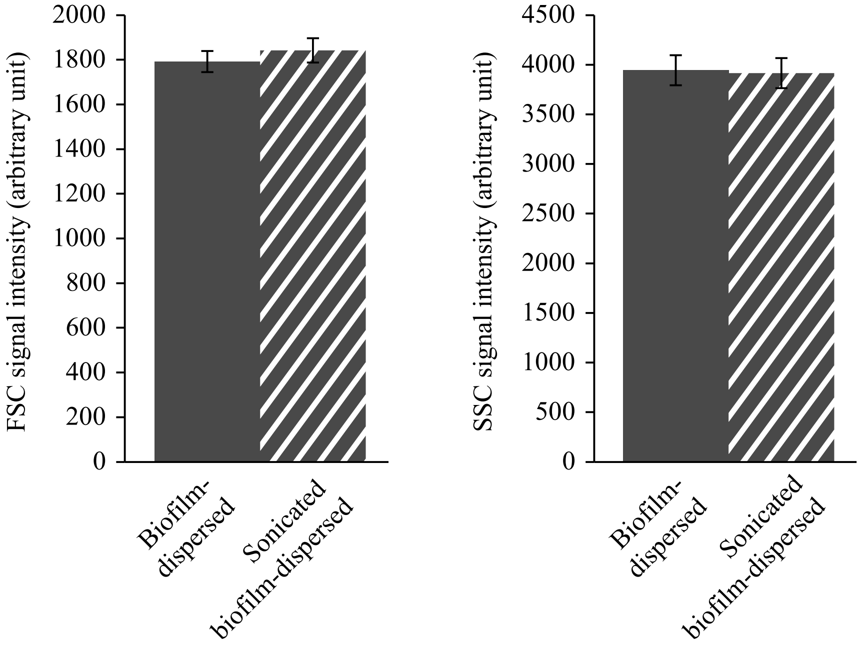

Supplementary Figure 3: Biofilm-dispersed bacteria did not comprise aggregates. Flow-cytometry analysis of biofilm-dispersed *K. pneumoniae* bacteria, sonicated or not, based on size (Forward Scatter; FSC) and structure (Side Scatter; SSC). Results are presented as the means of signal intensity for each channel. Each value is the mean of three independent experiments. Values represent mean ± s.e.m ($n = 3$). Statistics: non-parametric Mann-Whitney test.

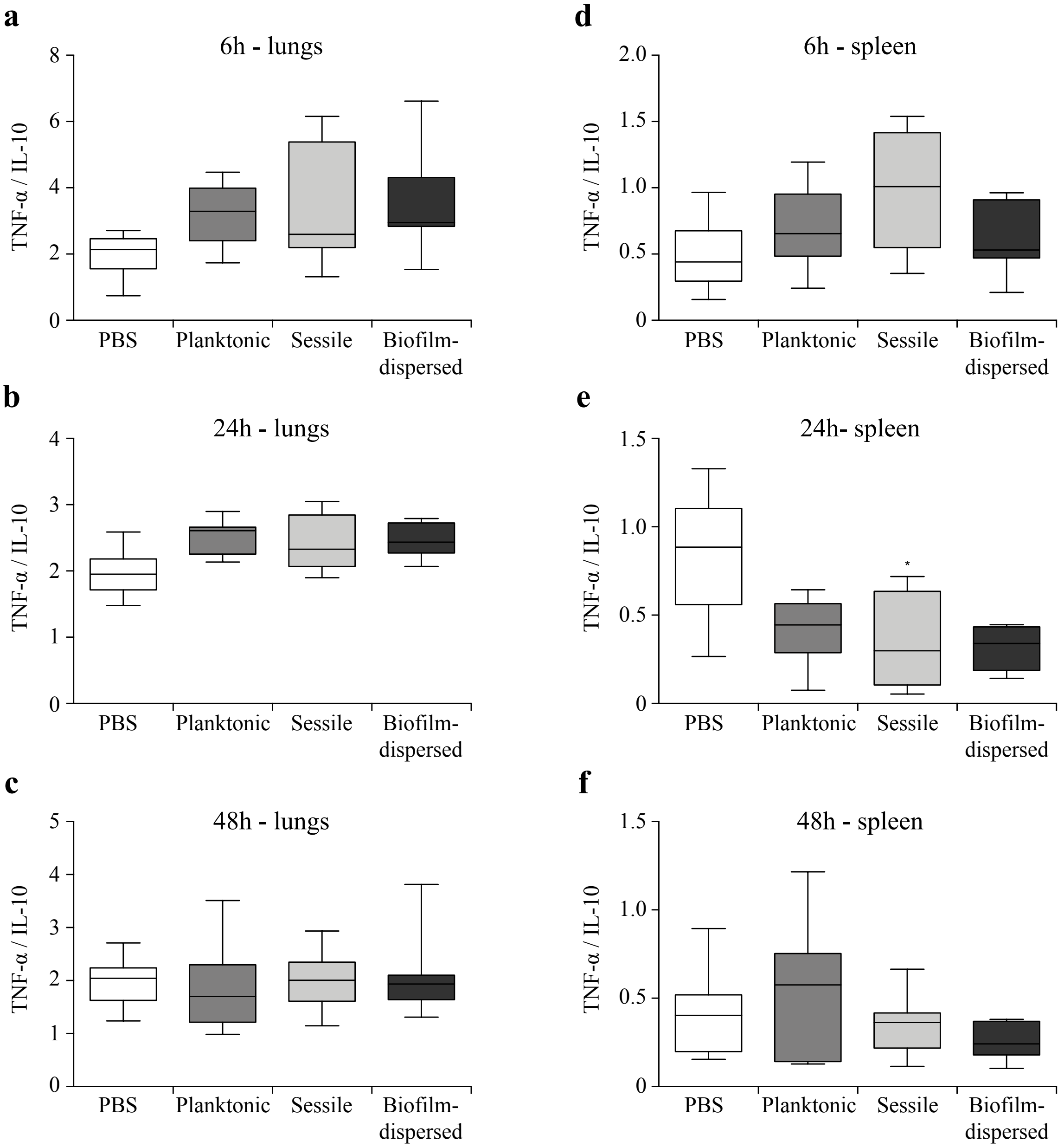

Supplementary Figure 4: TNF-α/IL-10 ratio calculated for each animal from cytokine levels detected in lungs (a-c) and spleen (d-f) at time points of 6h (a and d), 24h (b and e) and 48h (c and f) after inoculation. Boxplots reflect TNF-α/IL-10 ratio (n=8). The values are displayed as box-and-whiskers plots with interquartile range, with the top portion of the box representing the 75th percentile, and the bottom portion representing the 25th percentile. The horizontal bar within the box represents the median. Statistics: non-parametric Kruskal-Wallis with Dunn's multiple comparison test or Tukey's multiple comparison test for ELISA experiments: *, $p < 0.05$ in comparisons performed to the non-infected control (PBS).

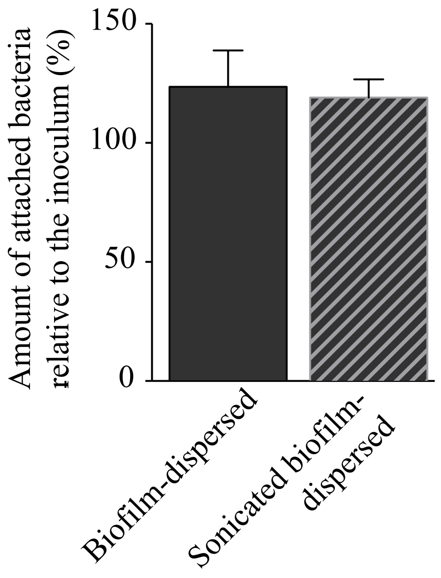

Supplementary Figure 5: Disruption of the potential aggregates by 3x5 min of sonication had no impact on the colonization capabilities of biofilm-dispersed bacteria. The glass surface colonization was assessed in the absence of chloramphenicol. Results are presented as the percentages of adherent bacteria after 3h of incubation compared to the CFU in the inoculum. Values represent mean ± s.e.m ($n = 3$). Statistics: non-parametric Mann-Whitney test

Supplementary Movie

Supplementary Movie 1: The kinetics of colonization of a glass surface in a context of competition (biofilm-dispersed bacteria versus planktonic bacteria) was monitored by confocal microscopy for 3h (one acquisition every 20 min). Cells derived from biofilm-dispersed and planktonic bacteria were distinguished by their specific fluorescent color: bacteria from planktonic cultures were tagged in green (GFP), and bacteria from biofilm-dispersed populations were tagged in red (mCherry). Images represent the first optical section of the z-stack from the surface.

Supplementary Table

Supplementary Table 1. Lewis acid–base and hydrophobicity surface characteristics

Growth conditions	% Affinity to solvant (MATS)					
	Chloroform	Hexadecane	Diethyl ether	Hexane	Ethyl acetate	Decane
Exponantial planktonic	21.21±0.43	8.43±0.56	12.06±0.50	7.36±0.47	0.59±0.55	8.65±0.40
Biofilm-dispersed	21.43±0.36	4.99±0.46	12.58±0.23	6.83±0.31	1.84±0.55	6.59±0.70

Whatever the solvent, the affinity to solvent was not significantly different (p<0.05) between the samples "exponantial planktonic" and "biofilm-dispersed". Statistics: non-parametric Mann-Whitney test.
